# Cyberbiosecurity for Biopharmaceutical Products

**DOI:** 10.3389/fbioe.2019.00116

**Published:** 2019-05-31

**Authors:** Jennifer L. Mantle, Jayan Rammohan, Eugenia F. Romantseva, Joel T. Welch, Leah R. Kauffman, Jim McCarthy, John Schiel, Jeffrey C. Baker, Elizabeth A. Strychalski, Kelley C. Rogers, Kelvin H. Lee

**Affiliations:** ^1^National Institute for Innovation in Manufacturing Biopharmaceuticals, Newark, DE, United States; ^2^Material Measurement Laboratory, National Institute of Standards and Technology, Gaithersburg, MD, United States; ^3^Office of Biotechnology Products (OBP), Center for Drug Evaluation and Research (CDER), U.S. Food and Drug Administration, Silver Spring, MD, United States; ^4^Information Technology Laboratory, National Cybersecurity Center of Excellence, National Institute of Standards and Technology, Gaithersburg, MD, United States; ^5^Office of Advanced Manufacturing, National Institute of Standards and Technology, Gaithersburg, MD, United States

**Keywords:** cyberbiosecurity, cybersecurity, biopharmaceutical manufacturing, engineering biology, cell therapy, gene therapy, supply chain

## Abstract

Cyberbiosecurity is an emerging discipline that addresses the unique vulnerabilities and threats that occur at the intersection of cyberspace and biotechnology. Advances in technology and manufacturing are increasing the relevance of cyberbiosecurity to the biopharmaceutical manufacturing community in the United States. Threats may be associated with the biopharmaceutical product itself or with the digital thread of manufacturing of biopharmaceuticals, including those that relate to supply chain and cyberphysical systems. Here, we offer an initial examination of these cyberbiosecurity threats as they stand today, as well as introductory steps toward paths for mitigation of cyberbiosecurity risk for a safer, more secure future.

## Introduction

Cyberbiosecurity is an emerging discipline encompassing vulnerabilities and corrective measures needed to address the unique risks existing at the intersection of cybertechnology and biotechnology. An early, inclusive definition of cyberbiosecurity is “understanding the vulnerabilities to unwanted surveillance, intrusions, and malicious and harmful activities which can occur within or at the interfaces of comingled life and medical sciences, cyber, cyber-physical, supply chain and infrastructure systems, and developing and instituting measures to prevent, protect against, mitigate, investigate, and attribute such threats as it pertains to security, competitiveness and resilience” (Murch et al., 2018).

To place context around the area of *cyberbiosecurity*, it is worth reviewing the established terms that contribute to this emerging discipline. *Cybersecurity* considers the security of digital information that is propagated and stored through networks of connected electronic devices (Lord, 2019). In general, *biosecurity* refers to the threat to living organisms and the environment due to exposures to biological agents, such as pathogens, whether occurring naturally or intentionally created (Institute of Medicine and National Research Council, 2006). A *cyber-biological interface* results when biological information is measured, monitored, or altered, and converted to digital information, or in the reverse, when digital information is used to manipulate a biological system. Similarly, a *cyber-physical interface* occurs when a physical mechanism is controlled or monitored by a digital means, such as the computer controlled mixing speed of a bioreactor. Importantly, cyber-physical interfaces may alter biological properties, blurring the lines of individualized definitions. Our intent in this publication is not to further refine the definition of cyberbiosecurity, as we believe that is best done through ongoing dialog within relevant stakeholder communities. Therefore, we rely on a working understanding of cyberbiosecurity as stated by (Peccoud et al., 2017), in referring to “the new risks emerging at the frontier between cyberspace and biology.” For the purposes of this paper, we focus on cyberbiosecurity for the manufacture of biopharmaceuticals, to raise awareness of the existing risks that will be compounded through innovation in both the emerging types of biologically-manufactured therapies and the increasingly-automated processes used to develop and manufacture them.

The biopharmaceutical industry contributes nearly one trillion dollars to the U.S. economy, and has been highly successful in industrializing biotechnologies to produce biologic therapeutics (PhRMA, 2017). Biopharmaceutical products, or biologics, use engineered biological systems as platforms to manufacture therapeutic products to prevent or treat a variety of health conditions, such as cancer, diabetes, autoimmune disorders, and microbial infections. These products include vaccines, traditional protein therapeutics, such as monoclonal antibodies, as well as emerging biotechnologies, such as cell and gene therapies.

Although the processes differ in how various classes of therapeutics are manufactured, in each process, information flows repeatedly between biological information (i.e., genetic) and cyber (i.e., digital) information. Securing this information flow through thoughtful assessment of vulnerabilities and threats for biopharmaceutical manufacturing is critical for public health, economic security, and national security. The focus of this publication is to illuminate these vulnerabilities and threats to encourage the broad stakeholder community to work toward the development of appropriate risk mitigation strategies, both for the current state-of-the-art and for the emerging technologies that represent the future state of the industry. Novel threats to the security of biological and related information along interfaces relevant to human health and manufacturing processes will continue to emerge as innovation progresses.

The interface of biological and digital information in biomanufacturing creates two primary concerns in evaluating cyberbiosecurity vulnerabilities, that recur throughout multiple processes in the end-to-end workflow (see Figure 1, Peccoud et al., 2017). The first concern is the nature of the biological manufacturing platform, as information contained in biological systems is subject to both evolution and context in ways that may not be well-understood or predictable. The variation that biological systems introduce in manufacturing presents risks for product consistency. The industry has developed extensive bioprocess control strategies and release testing to mitigate risks for established classes of biotherapeutics to ensure consistent product with minimal lot-to-lot variability. However, this biological variation presents challenges for innovating flexible scaling of existing large-batch processes. The issue of inherent biological variation is a critical challenge in the manufacture of emerging classes of gene and cellular therapies where typical small-batch manufacturing across a wider diversity of product types precludes the reliance on large historical data sets to allow identification of subtle process deviation. For these small-batch products, subtle genetic deviation during cellular expansion steps may be magnified *in vivo* due to differences between the host and the patient.

The second area of concern is the integrity of the data associated with the biopharmaceutical manufacturing process, including data related to supply chain and cyberphysical systems. Biopharmaceutical manufacturers are complex organizations that rely on technology as part of daily operations to tightly monitor and control biopharmaceutical production processes. The notion of a digital thread, which refers to data that follows a product and informs decisions throughout its life cycle, can be applied to the biopharmaceutical industry (Wang, 2018). The digital thread of the manufacturing of biopharmaceuticals includes data that support the development and scale up of the manufacturing process, clinical data, post-approval data, and the equipment used to manufacture the product. As the number of interconnected devices and systems that inform digital threads increases, cybersecurity vulnerability increases, because one vulnerable device can result in a threat that compromises a single point, or an entire process, system, or supply chain. Further, as a result of greater dependence on automation and decentralized manufacturing, the security of information transfer from site to site is critical to ensure the efficacy of the production process. While many cybersecurity concerns related to biopharmaceutical processes can be mitigated by existing best practices, standards, and regulations, the additional complexities at the cyber-biological interfaces during biopharmaceutical manufacturing processes, described below, warrant further examination.

The relevant stakeholder communities should establish a means of identifying and assessing the potential new vulnerabilities and threats, toward the development of effective risk mitigation strategies. For example, the NIST Framework for Improving Critical Infrastructure Cybersecurity is a voluntary, standards-based approach for identifying and protecting assets and systems, and detecting, responding to, and recovering from cyber intrusions (NIST, 2018). While the framework was originally developed for critical infrastructure systems where it has been widely adopted since its introduction in 2014, its focus on business drivers for cybersecurity risk assessment and practices makes it broadly applicable to many industries.

To further encourage the community's consideration of cyberbiosecurity vulnerabilities and mitigations, we include insights into the development of current cybersecurity best practices and guidance for medical devices, as a useful model for the path forward for a best-practices risk-mitigation framework for cyberbiosecurity for biopharmaceutical manufacturing. It is our hope that current biopharmaceutical industry practices can inform risk-mitigation for emerging classes of biotherapeutics and innovative production platforms for established classes of biotherapeutics. Current practices may also illuminate parallel considerations related to cyberbiosecurity in other biomanufacturing sectors and applications, such as synthetic biology approaches to the production of commodity chemicals and biofuels.

## Risks Associated with the Biological Manufacturing Platform in Biopharmaceutical Manufacturing Workflows

While best practices for cybersecurity apply to biopharmaceutical manufacturing, biological systems present unique vulnerabilities in production processes. Cyberbiosecurity vulnerabilities may be considered with regard both to using an engineered biological system as the manufacturing platform, as is the case for protein therapeutics, and for products that are themselves an engineered biological system, as for cellular therapies. The dynamic nature of genetic information that aids survival in natural environments poses challenges in engineering and manufacturing settings. For example, some change in the genetic information of a cell population is unavoidable during expansion and growth in a bioreactor, so biomanufacturing processes must contend with heterogeneous populations of cells that may yield a heterogeneous product, whether biomolecular or cellular. The ability of biological systems to alter the content and expression of their genetic information presents significant complexity for biopharmaceutical manufacturing unique to those posed by cyber-systems that must be considered in strategies for cyberbiosecurity risk mitigation.

### Challenges of Genetic Information

Two fundamental distinctions between digital and biological information are relevant in considering the cyber-biological interface during the end-to-end biopharmaceutical manufacturing process. First, genetic information evolves naturally when replicated. Mechanisms that drive natural changes in DNA sequence include mutation, recombination, horizontal gene transfer, and others. Second, the expression of this information can change depending on how an organism senses and responds to its environment. This dependence on context, which encompasses all aspects of the system in which the genetic information exists, cannot always be predicted. The same sequence of DNA may have dramatically different consequences for function depending on surrounding DNA sequences, intra- and inter-molecular interactions within the cell, and extracellular conditions. Thus, the impact of changes, whether due to natural “drift” or through malicious introduction, is difficult to predict, detect, and mitigate.

### Protein Therapeutics

State-of-the-art biomanufacturing of protein therapeutics uses engineered mammalian cells as the manufacturing platform. One notable example is Chinese hamster ovary (CHO) cells used as the host cell system (Jayapal et al., 2007). To better assess potential vulnerabilities at the cyber-biological interface in this process, we consider the flow of genetic information in a typical biomanufacturing workflow.

The security of the genetic information at the cyber-biological interface is assured initially through the integrity of the nucleic acid used to transfect a cell line. Programmable DNA synthesizers and sequencers specify and confirm the DNA sequence that is then stably transfected into host cells for cell line development. This process effectively transfers digital information into a “genetic thread” that parallels the digital thread of the manufacturing process. A selection of clonal cells with desired phenotypes for yield and stability are then passaged under defined conditions to produce master cell banks, which are passaged further to produce working and production cell banks. Throughout these workflows, consistent cell culture expansion protocols are used to achieve consistent context for the genetic information, with the intent of minimizing natural mutations. Contextual security of the genetic information during production is also maximized through well-defined process control strategies. This context includes bioreactor growth conditions, such as feeding strategy, dissolved oxygen concentration, gas flow, sparge rates, pH, and temperature. Cell populations that exhibit genetic instability during bioreactor growth are identified through deviations from established process parameters, so that processes can be aborted at early stages, and there is no risk to product quality. Genetic stability across the expanded cell populations is also monitored for transgene sequence and copy number, including the testing of post-production cell banks to ensure data across the full thread of genetic information. As the natural evolution of the cells during expansion cannot be reversed, the security of the master cell banks is critical to ensure the consistency of the product through its lifecycle, and redundancies are built into storage strategies to guard against any single failure mode.

At the state of the art, the industry is mitigating risks associated with the uncertainty in product safety profiles due to natural variation or contamination in the biological system, through extensive control and quality assurance strategies, following established best practices and rigorous regulatory guidance. Furthermore, as facility access is currently managed to ensure both protection of trade secrets and compliance with current U.S. FDA Good Manufacturing Practices regulations, it is difficult to imagine scenarios where malicious or adventitious acts on bioprocess workflows would go undetected for established manufacturing facilities producing protein therapeutics through large batch processes. However, a malicious intrusion increases uncertainty at the cyber-biological interface and could trigger batch losses, with significant economic impacts for the industry and could potentially result in drug shortages (Castellanos and Janofsky, 2018).

During the production of protein therapeutics, cyberbiosecurity vulnerabilities exist at each point where genetic information is stored, expressed, replicated, or monitored through cyber or cyber-physical systems. A simple example is the storage of master cell banks in a freezer with networked alarm and temperature monitoring systems, where failure in the network can introduce uncertainty in the viability of the master cell bank. A more malicious variation of this simple scenario is a cyber-intrusion that corrupts the digital record that documents the storage conditions for the master cell bank. In both cases, the uncertainty of the cells' viability presents a vulnerability, even if the actual impact on the stored cells was negligible.

A more complex example of a dynamic cyber-biological interface is a perfusion bioreactor. In this process, flow rates of media into the reactor and biomass removal out of the reactor are balanced to maintain a desired cell density within the bioreactor. The cell density is optimized for process yield and growth rate is controlled through parameters such as nutrient limitation (Bielser et al., 2018). The cyberphysical components of the system control media and biomass flow rates, which in turn constrain cellular growth rate and product yield. Thus, the vulnerabilities associated with the cyberphysical control system propagate into vulnerabilities in the biological output of the process.

As typical workflows for the production of protein therapeutics are fully established and industrialized, many of the risks are mitigated by current manufacturing practices. However, this discussion is intended to prompt a systematic evaluation of vulnerabilities and threats at the cyber-biological interfaces for these processes, both to reduce remaining vulnerabilities to malicious acts, and to inform risk-mitigation strategies for less-industrialized manufacturing workflows.

### Emerging Classes of Biologic Therapies

Increasingly, engineered cells are themselves the therapeutic product, rather than simply serving as the biomanufacturing platform. For example, CAR-T cells (Androulla and Lefkothea, 2018) and engineered microbiome modulators (Garber, 2015) are members of a growing category of existing living therapeutics enabled by engineering biology methods. For these living therapeutics, as well as for *in vivo* gene therapies, the flow of genetic information occurs in both the production for the therapeutic agent, and within the patient. Each of the biosecurity considerations for protein therapeutics applies to living therapeutic modalities, but protein therapeutics benefit from decades of experience in production, as well as testing of product lot releases to identify, in principle, any relevant deviations in the flow of genetic information. Aside from unwanted physicochemical degradation, protein therapeutics cannot alter their own properties or respond to environmental context. Established process controls and quality assurances in protein therapeutic biomanufacturing should be adapted to address the emerging cyberbiosecurity needs of emerging novel modalities. However, emerging product modalities such as cellular and gene therapies convey alterations in genetic information that are intended to become self-replicating and expressed *in vivo*. These emerging therapies therefore pose additional safety concerns for patients that warrant further cyberbiosecurity evaluation of their manufacturing workflows, as well as pharmacovigilance at the patient level to monitor the integrity of the transferred genetic code.

### Future Therapeutic Modalities

Engineered cells from all domains of life, including prokaryotes, eukaryotes, and archaea, as well as synthetic systems, such as cell-free systems, may offer potential biomanufacturing platforms and products in industrial workflows. The ongoing evolution of biotechnology fueled by increasingly automated DNA design, read, and write capabilities, along with facile gene-editing platforms, such as CRISPR, TALENs, and zinc-finger nucleases will continue to create new cyber-biological interfaces and additional risks for both biosecurity and biosafety.

Proof-of-concept exists for designing genetic circuits that can be used to encode logic in bacteria and enable them to perform clinically-relevant functions (Brophy and Voigt, 2014). In principle, cells could be engineered using genetic circuits to treat a wide range of pathologies, including but not limited to autoimmune diseases, cancer, and viral infections (Piñero-Lambea et al., 2015; Xie and Fussenegger, 2018). Computational methods that leverage principles from electronic design automation have been employed for the design and optimization of these genetic circuits (Nielsen et al., 2016). Genetic circuit design software, such as that offered by Teselagen, can automatically generate machine-readable synthesis instructions. Any processes similar to these, which involve the transfer of information between digital and biological forms, are potential points of vulnerability. While current biomanufacturing processes may be difficult to disrupt without detection, fully automated, distributed and “on-demand” biomanufacturing workflows of the future may make it possible to use malicious cyber-intrusions to corrupt the design, reading, and writing of DNA sequences to produce pathogenic, self-replicating entities that pose both biosecurity and biosafety hazards. Although these risks are still emerging, the rapid pace of innovation dictates that it is not too early to consider the cyberbiosecurity implications of such capabilities. The National Academies of Sciences, Engineering, and Medicine have recently assembled a committee to consider strategies on Safeguarding the Bioeconomy that is expected to contain an analysis of the unique elements of the biotechnology economy that will consider whether specific features of the bioeconomy may require innovative cybersecurity solutions.

### Future Cyber-Biological Interfaces Enabled by Artificial Intelligence

Digital data may become increasingly similar to biological data, in that digital data may become more dynamic and dependent on its context, especially considering the expanding capabilities of artificial intelligence (AI) and the increasingly widespread implementation of machine learning algorithms. Looking forward, computers and biology in the same control loop is an emerging area that could introduce new cyberbiosecurity vulnerabilities as AI and machine learning become more mainstream. While current AI capabilities are mostly associated with passive learning, systems capable of active learning and neural networks are currently being developed for many different applications (Murphy, 2011; Lou et al., 2014; Angermueller et al., 2016; Jamali et al., 2016; Feltes et al., 2018). As artificial intelligence finds increasing application in biomanufacturing and transitions from completely dependent to semiautonomous to completely autonomous, a full assessment of vulnerabilities and threats should include strategies for mitigation. With each advance, cybersecurity and cyberbiosecurity may more fully approach a single, unified discipline.

## Cyberbiosecurity, Process Control and Quality/Risk Management

Biopharmaceutical manufacturing relies on complex technology as part of daily operations to tightly monitor and control biological production processes. Many of the failure modes in biopharmaceutical manufacturing are foundationally similar to those of other manufacturing modalities, and existing best practices in cybersecurity should be incorporated to mitigate those risks. The complexity of the digital thread arising from the biological component of the manufacturing process for biopharmaceuticals introduces additional risks that can impact product quality. We, therefore, propose that cyberbiosecurity should also be considered as a failure mode in the development of a manufacturing control strategy, and in maintenance of the validated state.

Physicochemical and biophysical data related to a biopharmaceutical product, for example, is generated throughout its lifecycle, detailing early generation products, reference material qualification, stability testing, and release strategies. Biological License Applications summarize this data through submission of a common technical document to regulatory authorities. The data originator must safeguard both raw and processed forms of this data for extended periods, typically years, for trending, re-evaluation, and comparison to support future comparability studies.

As mentioned in the above section, the aftermath of a cyberbiosecurity failure can have a significant impact on supply of medicines and on patient health. For example, many biopharmaceutical products are high-potency, low-volume operations, with a year or more of inventory generated in a single lot. A failure in such a manufacturing process would dangerously deplete the supply of that product. Furthermore, many biopharmaceutical processes contain non-compressible timelines (e.g., expansion cultures or hydrodynamic limitations), so timely recovery from a cyberbiosecurity failure could be difficult, especially in a high-utilization, multi-product plant. Patients that rely on biopharmaceuticals can be especially impacted by shortages or recalls because it is not uncommon for biopharmaceutical products to be presented through extended courses of therapy that have negative clinical consequences if interrupted.

### Pharmaceutical Quality Management Systems

Pharmaceutical Quality Management Systems (QMS) are implemented to deliver products with appropriate quality attributes, establish and maintain a state of control, and facilitate continual improvement in manufacturing processes. By necessity, a QMS assumes that valid monitoring and assessment of the process are in place. A cyberbiosecurity breach has the potential to “break” an integrated QMS. If fundamental QMS activities, such as in-process and finished product analysis, inventory management, document management, change control, lot disposition, corrective actions, and preventative actions, were compromised by a cyberbiosecurity breach, biopharmaceutical manufacturing operations would have to either be shut down or subject to detailed, manual review, and assessment. This practice at best increases costs and human-sourced variation, and at worst compromises the quality of the product produced.

A QMS anticipates and detects special cause variation in the context of common cause variation. A cyberbiosecurity failure could present itself as an unanticipated or undetected special cause failure (e.g., an adventitious or malicious alteration or contamination of the data stream), or could cloud understanding of common cause variation (e.g., system decay or continuous improvement of operations). In particular, undetected cyberbiosecurity “contaminations” could be particularly worrisome. An undetected cyberbiosecurity failure could manifest in, for example, incorrect test results or expiry dates, incorrect process control loops and algorithms, inappropriate conduct of maintenance in the plant, or even disruption through presentation of false failures during inspection by regulators. For these reasons, assessment of cyberbiosecurity vulnerabilities should be built into a lifecycle control maintenance plan assuring the validated state. Different manufacturing processes may require different risk-based cyberbiosecurity measures to address different threats and vulnerabilities, however they should all be framed by the QMS.

Continuous improvement in biopharmaceutical manufacturing is predicated upon comparability exercises. Cyberbiosecurity failures that compromise the integrity of comparability can prevent continuous improvement and deployment of new technologies or manufacturing sites.

### Manufacturing Process Control and Product Quality

Manufacture of traditional biopharmaceutical products, such as protein therapeutics, has a high level of residual uncertainty, making this type of manufacturing particularly vulnerable to cyberbiosecurity failure modes. Increasingly, biopharmaceutical manufacturers are employing a greater dependence on process analytical technologies, automation, and distributed and integrated control systems, with fewer manual interventions. This shift decreases human factor-related failure, but increases the likelihood of cyberbiosecurity-related failures for biopharmaceuticals.

Because engineered biological systems are used as the manufacturing platform, control of the product is a function of control and evaluation of multiple critical quality attributes (CQAs) and process parameters rather than direct measurement of clinically relevant mechanistic functions. Process control strategies monitor common and special cause variability, sort variations into relevant (signal) and indeterminate (noise), and trigger corrective actions. The acts of monitoring, sorting, and communicating corrective actions are vulnerable to cyberbiosecurity threats. These failure modes can lead to special cause errors, which can subsequently lead to false or misleading signals, or undetected or uncommunicated process failures.

As those in process development increase use of process analytical technology (e.g., on-line/at-line testing) and move toward real-time release, there is less opportunity for detection and mitigation of a cyberbiosecurity breach. For example, processes depend more upon validated clearance of process-specific contaminants (e.g., DNA, viruses, host cell proteins, residual solvent, etc.) rather than lot-to-lot testing. A compromise to the validated processing envelope in the form of a cyberbiosecurity breach could impact product quality and safety because the assumption supporting clearance established during process validation would no longer be valid. A shift in process control toward real-time release could increase the possible impact of a cyberbiosecurity failure compared to lot-to-lot release testing. This is not to say that real time release practices should be avoided but rather that dynamic risk assessment modeling is crucial to understanding these advanced control strategies.

### Manufacturing Supply Chain Considerations

Biopharmaceutical manufacturing frequently uses reagents or materials with few alternative vendors. The risk and impact of a cyberbiosecurity failure within the supply chain or at a key vendor could have an unanticipated, negative impact on the assurance of a consistent supply of high-quality biopharmaceuticals. A second supply chain consideration is for the biopharmaceutical product itself. These products are often sterile parenterals with cold chain conformance requirements. Indirect adventitious or malicious cyberbiosecurity attacks to maintenance of sterile operations or to the cold chain could lead to loss of product or, worse, could compromise patient safety or efficacy.

### Cybersecurity for Medical Devices as a Model for Developing Risk Evaluation and Mitigation Strategies for Cybersecurity Vulnerabilities in Biopharmaceutical Manufacturing

The medical device industry faced a similar challenge, as medical devices create a cyber-biological interface with direct patient impact. As devices become increasingly interconnected, cybersecurity concerns for medical devices, such as device access and security of information and data, drove community engagement to develop best practices to address these concerns. Cybersecurity specifically refers to the protection of computer systems, including hardware, software, and data, from unauthorized access, theft, damage, disruption or misdirection. A medical device itself has hardware, software, and data that could potentially be compromised after a cybersecurity attack. The community engaged with the FDA Center for Devices and Radiological Health (CDRH) to systematically evaluate risks at all points in the device life cycle and then to develop best practices to mitigate these risks. As a result of these efforts, CDRH has released three Guidance for Industry documents [FDA., 2014, 2016, 2018 (draft)], and hosted four public workshops where discussion of medical device technology, device regulation, policy gaps, and best practices was welcomed. Similarly, community engagement between all stakeholders including industry and regulators, could lead to the development of best practices for cyberbiosecurty in the biopharmaceutical industry.

## Conclusions

Biopharmaceutical products have had a substantial positive impact on public health. With the increasing digitalization of information related to such products and how they are manufactured, it becomes important to consider potential impacts from cyberbiosecurity-related threats. Detected intrusions will trigger the need for investigation and mitigation within a robust quality management system. Among the potential impacts are:

- Economic loss to the industry due to a manufacturing process out of specifications, poor product quality, or loss of confidence in the integrity of the process.
- Patient and public health impacts due to ineffective, dangerous, or lost production batches, most notably for autologous therapies, such as CAR-T.
- Exposure of employees to harmful agents, for example, through the deliberate introduction of a pathogen into manufacturing process.
- Inability to respond rapidly to emergent public health threats.

Therefore, analysis is warranted to identify and mitigate the unique cyberbiosecurity risks and failure modes in the biopharmaceutical industries. Current best practices from industrial manufacturing and state-of-the-art cybersecurity could serve as a starting point to safeguard and mitigate against cyberbiosecurity threats to biomanufacturing.

Given the importance of the issues raised by cyberbiosecurity risks, ecosystem-wide coordination and communication to develop a more comprehensive understanding of the field as well as appropriate mitigation strategies are needed. One possible path forward may be to explore the use of NIST's Framework for Improving Critical Infrastructure Cybersecurity to manage risks introduced by vulnerabilities and threats unique to biological systems. The framework could potentially be adapted or profiled with input from stakeholders to include relevant standards, guidelines, and best practices to manage cyberbiosecurity risks for biomanufacturing organizations of all scales. The framework could allow businesses and organizations to develop their own unique profile to address risk appetite, mission priority, budget, and resource constraints within the scope of their requirements, objectives, and desired outcomes. A follow-on publication from NIST provides a manufacturing-specific roadmap for reducing cybersecurity risk that may provide additional guidance to the biomanufacturing community (Stouffer et al., 2017).

Cyberbiosecurity concerns should be a part of modern, risk-based, quality management systems and should be considered in the development and maintenance of process control strategies throughout the product life cycle. Education and awareness of existing best practices for cybersecurity of manufacturing systems is essential for personnel involved in any stage of these processes. Creating standard practices to fully incorporate cyberbiosecurity awareness into every stage of the biomanufacturing process can lead to a more secure supply of safe, life-saving medicines, ultimately improving lives through a healthy society, and strong economy.

## Author Contributions

JLM, KR, and KL contributed to all sections of the manuscript. JW and JB led the writing of the section on biopharmaceutical manufacturing and made comments and edits on other sections. JR, ER, LK, JM, JS, and ES led the writing of the section on biopharmaceutical products and made comments and edits on other sections.

## Disclosure

This publication reflects the views of the author and should not be construed to represent FDA's views or policies. Certain commercial entities, equipment, products, or materials may be identified by name in order to describe an experimental procedure or concept adequately. Such identification is not intended to imply special status or relationship with NIST or recommendation or endorsement by NIST; neither is it intended to imply that the entities, equipment, products, or materials are necessarily the best available for the purpose.

### Conflict of Interest Statement

The authors declare that the research was conducted in the absence of any commercial or financial relationships that could be construed as a potential conflict of interest.
